# Supplementary material for: Enzymatic Activity and Microbial Diversity of Sod-Podzolic Soil Microbiota Using 16S rRNA Amplicon Sequencing following Antibiotic Exposure
Source: Antibiotics (Basel). 2021 Aug 12;10(8):970. doi: 10.3390/antibiotics10080970 (PMC8388902; doi:10.3390/antibiotics10080970)
Supplement: Supplementary file 1 [file antibiotics-10-00970-s001.zip › antibiotics-1248244-supplementary.pdf]

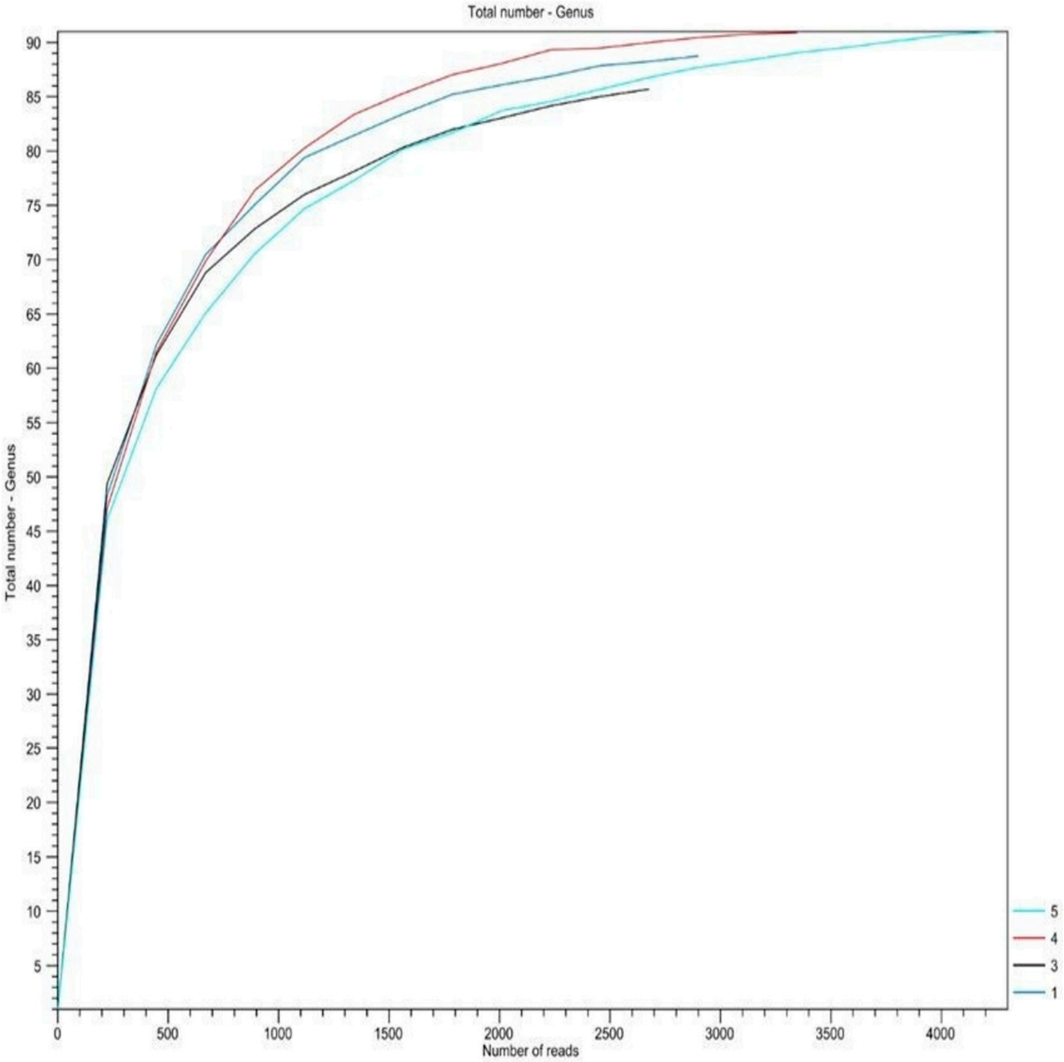

Figure S**1.** The ratio curve between the bacterial community diversity at the genus level and selective effort. 1: untreated soil sample (control); 3: soil treated with benzylpenicillin; 4: soil treated with oxytetracycline; and 5: soil treated with tylosin.
